# Performance of potentially inappropriate medications assessment tools in older Indian patients with cancer

**DOI:** 10.1002/cam4.6797

**Published:** 2024-01-06

**Authors:** Sharath Kumar, Renita Castelino, Abhijith Rao, Shreya Gattani, Anita Kumar, Anupa Pillai, Arshiya Sehgal, Pallavi Rane, Anant Ramaswamy, Ratan Dhekale, Jyoti Krishnamurthy, Shripad Banavali, Rajendra Badwe, Kumar Prabhash, Vanita Noronha, Vikram Gota

**Affiliations:** ^1^ Department of Clinical Pharmacology Advanced Centre for Treatment, Research and Education in Cancer, Tata Memorial Centre Navi Mumbai India; ^2^ Department of Medical Oncology Tata Memorial Hospital Mumbai India; ^3^ Department of Statistics Advanced Centre for Treatment, Research and Education in Cancer, Tata Memorial Centre Navi Mumbai India; ^4^ Homi Bhabha National Institute Mumbai India; ^5^ Department of Surgical Oncology Tata Memorial Hospital Mumbai Maharashtra India

## Abstract

**Background:**

Polypharmacy and potentially inappropriate medication (PIM) use are common problems in older adults. Safe prescription practices are a necessity. The tools employed for the identification of PIM sometimes do not concur with each other.

**Methods:**

A retrospective analysis of patients ≥60 years who visited the Geriatric Oncology Clinic of the Tata Memorial Hospital, Mumbai, India from 2018 to 2021 was performed. Beer's‐2015, STOPP/START criteria v2, PRISCUS‐2010, Fit fOR The Aged (FORTA)‐2018, and the EU(7)‐PIM list‐2015 were the tools used to assess PIM. Every patient was assigned a standardized PIM value (SPV) for each scale, which represented the ratio of the number of PIMs identified by a given scale to the total number of medications taken. The median SPV of all five tools was considered the reference standard for each patient. Bland–Altman plots were utilized to determine agreement between each scale and the reference. Association between baseline variables and PIM use was determined using multiple logistic regression analysis.

**Results:**

Of the 467 patients included in this analysis, there were 372 (79.66%) males and 95 (20.34%) females with an average age of 70 ± 5.91 years. The EU(7)‐PIM list was found to have the highest level of agreement given by a bias estimate of 0.010, the lowest compared to any other scale. The 95% CI of the bias was in the narrow range of −0.001 to 0.022, demonstrating the precision of the estimate. In comparison, the bias (95%) CI of Beer's criteria, STOPP/START criteria, PRISCUS list, and FORTA list were −0.039 (−0.053 to −0.025), 0.076 (0.060 to 0.092), 0.035 (0.021 to 0.049), and −0.148 (−0.165 to −0.130), respectively. Patients on polypharmacy had significantly higher PIM use compared to those without (OR = 1.47 (1.33–1.63), *p* = <0.001).

**Conclusions:**

The EU(7)‐PIM list was found to have the least bias and hence can be considered the most reliable among all other tools studied.

## 1 INTRODUCTION

Cancer is a global health problem which affects millions of people each year. Despite advances in treatment, it is the second most common cause of death following cardiovascular diseases.^1^, ^2^ Being predominantly a disease of older patients, the burden of disease worldwide is likely to increase with increasing life expectancy.^3^ The incidence of cancer and age‐specific mortality rate increases parallelly with advancing age and a peak is noted at 80–84 years.^4^ Incidentally, comorbidities also increase with age, necessitating multiple medications in older cancer patients.^5^ Potential drug–drug interactions (DDI) in a polypharmacy scenario and relative contraindications in older patients can increase the risk of potentially inappropriate medication (PIM) use.^6^ PIMs in older adults are drugs that should be avoided due to their propensity to cause adverse reactions in this population, or having insufficient evidence to support their use when safer and effective therapeutic alternatives are available.^7^ In fact, a study from our center identified polypharmacy and PIM use as common problems associated with pharmacological interventions in older Indian patients with cancer.^5^ Inappropriate prescribing of medications in older adults is associated with negative outcomes including adverse drug reactions (ADRs), hospitalization and higher economic burden.^8^, ^9^, ^10^ The American Geriatrics Society Beers Criteria has advocated reduction in the number of medications and elimination of high‐risk medications for all older adults to mitigate PIM.^11^ Quite a few studies have assessed PIM use in older cancer patients.^12^, ^13^ Approximately 30% of older cancer patients are exposed to severe drug interactions and PIMs, and there are validated methods to assess PIM as a standard part of geriatric assessment.^13^, ^14^ The revised AGS Beers criteria (2019), STOPP/START criteria version 2 (2015), PRISCUS list (2010), Fit fOR The Aged (FORTA) (2018), and the European Union (EU) (7)‐PIM list are some of the well‐known, validated methods to assess PIM. However, there is broad heterogeneity in the identification of PIM with these tools. These tools may vary in specific medications or drug classes they identify as PIM, and some in their approach to categorizing medications (e.g., Beers criteria lists medications to avoid or use with caution, while FORTA categorizes them by appropriateness), thus highlighting the need for a universal consensus on this matter.^15^, ^16^, ^17^, ^18^, ^19^, ^20^ Importantly, the relative performance of these tools in identifying PIM in older Indian adults with cancer is largely unknown. Therefore, this study was designed to compare the validated screening tools for the assessment of PIM in older Indian patients with cancer and identify the most suitable tool for our practice.

## 2 METHODS

### 2.1 Study population

A retrospective analysis of medications that are potentially inappropriate for older adults with cancer was undertaken from June 2018 to August 2021 at the Tata Memorial Hospital, Mumbai, India. The study was approved by the Institutional Ethics Committee of the hospital. In India, elderly individuals have been defined in the National Policy for Older Person (1999) as people above 60 years of age. All elderly patients referred to the Geriatric Oncology Clinic at the hospital undergo complete geriatric assessment (CGA) including a comprehensive medication review by a multidisciplinary team comprising of medical oncologists, geriatricians, and clinical pharmacologists. Oncologists and geriatricians performed CGA, while the clinical pharmacologists performed a thorough medication reconciliation and identified PIM using various tools. The compiled data from the team was discussed during a joint clinic, and further recommendations were provided to the patient. Patients or caregivers who refused to undergo the geriatric assessment and those with an Eastern Cooperative Oncology Group performance status of four were excluded from this study.

### 2.2 Data compilation

As a routine practice in the geriatric clinic, patients are requested to provide a complete overview of their ongoing medications during the assessment. Electronic medical records and the online prescription system are reviewed for each patient. Thorough medication reconciliation will be carried out wherein actual medication use, including non‐prescription medicines, are verified with the patient. The use of medications from alternative systems are also documented but not included in the PIM assessment. In case of combination drugs, each constituent drug is counted separately. Anti‐cancer agents are not considered in the analysis; however, supportive care medications are evaluated for inappropriateness according to the tools. As a part of the geriatric assessment, the presence of various symptoms/syndromes that are necessary for PIM assessment, for example, insomnia, urinary incontinence, history of falls, dementia, etc. are also recorded. The duration and frequency of use of medication are also recorded as some tools identify PIMs based on the duration of medication use. The Cockcroft–Gault equation was used for the estimation of renal function in all patients. The data is prospectively entered on Statistical Package for the Social Sciences (SPSS v.23). The data entered between June 1, 2018 and August 6, 2021, was used for this study.

### 2.3 PIM identification

For each patient, PIMs are identified using the revised AGS Beers criteria (2019), STOPP/START criteria version 2 (2015), PRISCUS list (2010), FORTA (2018), and the European Union (EU) (7)‐PIM list. As a matter of practice, only PIMs identified as per the Beer's criteria are forwarded to treating physicians for consideration. A standardized PIM value (SPV) was assigned for each patient for each scale which represented the ratio of the number of PIMs identified by a given scale to the total number of medications taken. In the absence of a “gold standard” tool for PIM assessment, the median SPV of all five tools for each patient was considered the reference standard.

### 2.4 Statistical analysis

Agreement between each scale and the reference was carried out using Bland–Altman plots. The mean difference in the SPV between a given scale and the reference standard was a measure of the bias. The ±1.96 standard deviation of the bias estimate provided the upper and lower limits of agreement (LOA). Lin's concordance coefficient was also employed to quantify the level of agreement between SPV of each scale and the median SPV. This coefficient, designed for measuring the concordance or agreement between two continuous variables, considers both precision and accuracy.^21^ Descriptive statistics (Mean ± SD) was used to analyze continuous variables. Relationship between categorical variables such as sex, comorbidities (present/absent), number of medications (<5, ≥5) and the outcome PIM (yes/no) was determined using the univariate Chi‐square test followed by multiple logistic regression analysis. PIM use was considered as “yes” if any of the five tools identified at least one PIM. Statistical tests were carried out using R 4.1.2, IBM SPSS Statistics v.23 and GraphPad Prism 8.0.2.

## 3 RESULTS

### 3.1 Patient characteristics

**FIGURE 1:**
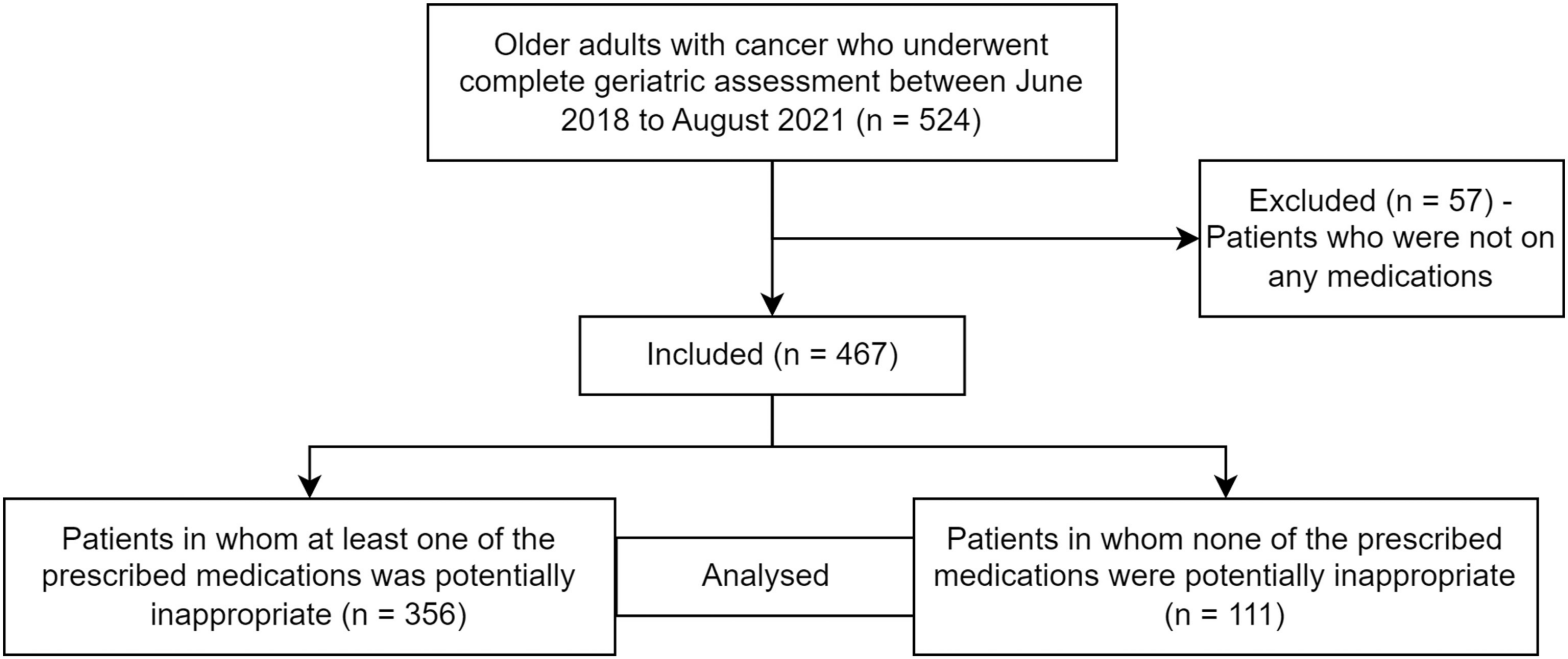
Schematic representation of patient selection, and analysis.

Out of the 524 patients who underwent CGA between the study period, 467 patients were included in the analysis of which 372 (79.65%) were male. The mean age of the patients was 70.0 ± 5.9 years. Hypertension (48.82%) was the most frequent comorbidity followed by diabetes (28.26%). Non‐Small Lung Cancer (NSCLC) was the most common primary cancer (37.68%). A schematic representation of patient selection process is shown in Figure 1. The demographic data of the participants is shown in Table 1.

**TABLE 1:** Patient demographics.

Parameters	*n* = 467 (*n*) (%)
Age (Mean ± SD)	70.00 ± 5.91
Sex	
Female	95 (20.34)
Male	372 (79.65)
Geriatric syndromes	
Insomnia	121 (25.91)
Constipation	139 (29.76%)
Urinary incontinence	31 (6.63)
LUTS	126 (26.98)
Dizziness	36 (7.70)
Pressure ulcers	3 (0.64)
Osteoporosis	19 (4.06)
Vision impairment	171 (36.61)
Hearing impairment	54 (11.56)
Ulcer or acidity	127 (27.19)
Comorbidities	
HTN	228 (48.82)
DM	132 (28.26)
COPD	64 (13.70)
Cardiovascular disease	81 (17.34)
Primary tumor diagnosis	
NSCLC	176 (37.68)
CA esophagus/ GEJ	95 (20.34)
CA head and neck	55 (11.77)
CA prostate	30 (6.42)
Other malignancies	111 (23.7)
Creatinine clearance (Mean ± SD)	68.21 ± 24.77

Abbreviations: CA, Cancer; COPD, Chronic Obstructive Pulmonary Disease; DM, Diabetes Mellitus; GEJ, Gastro‐Esophageal Junction; HTN, Hypertension; LUTS, Lower Urinary Tract Symptoms; NSCLC, Non‐Small Cell Lung Cancer; SD, Standard Deviation.

### 3.2 Performance characteristics of PIM tools

**FIGURE 2:**
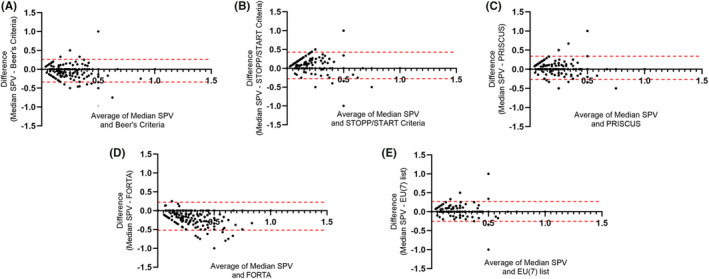
Bland–Altman plots displaying the agreement between each scale to reference standard or the median SPV. (A) Beer's Criteria, (B) STOPP/START criteria, (C) PRISCUS, (D) FORTA, (E) EU (7)‐PIM list. Bland–Altman plots the difference between the two measurements (median SPV & scale specific score) and the mean of the same. FORTA, Fit fOR The Aged; PIM, potentially inappropriate medication; SPV, standardized PIM value.

**FIGURE 3:**
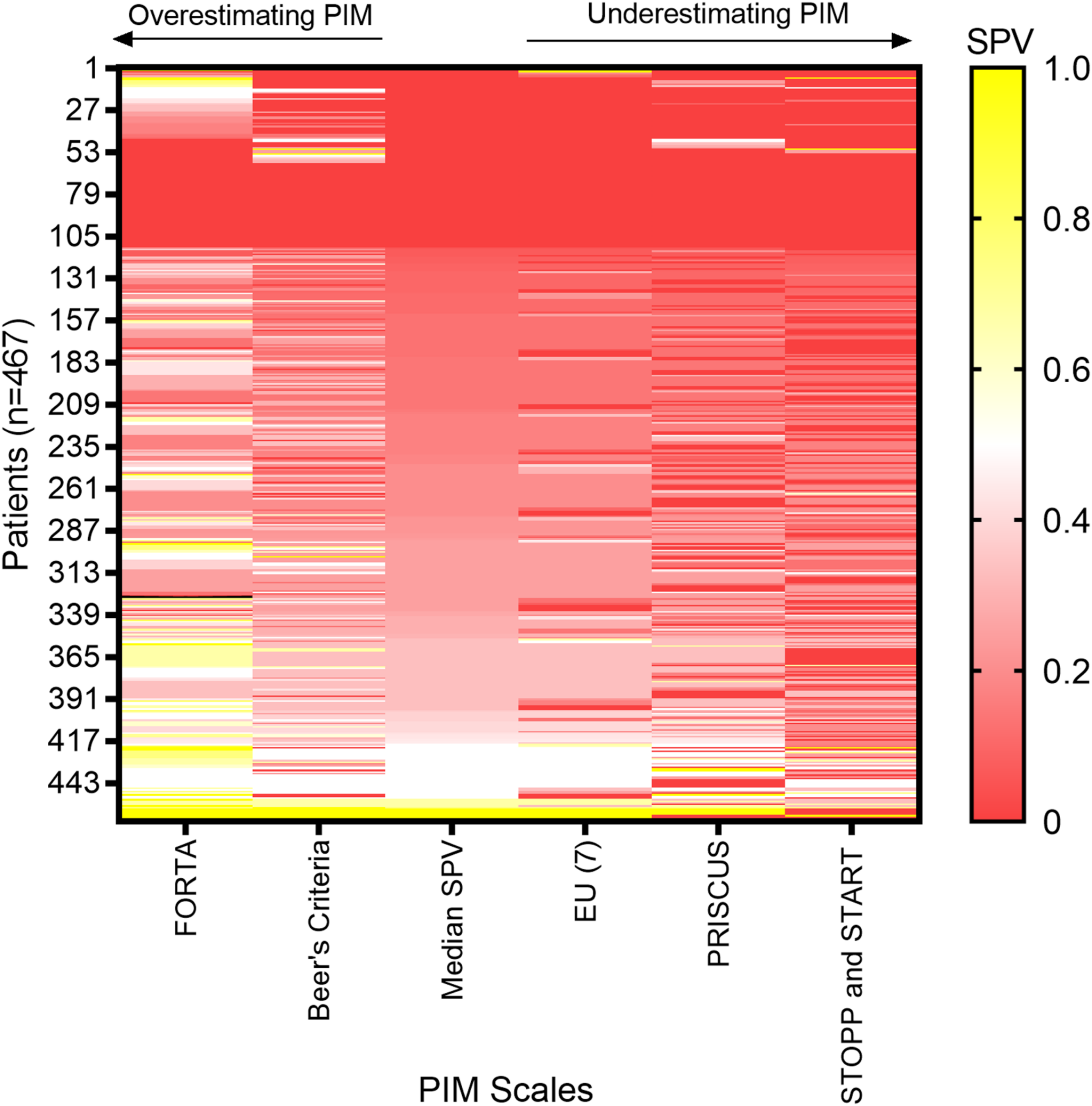
Heat map displaying the standardized PIM value (SPV) of each patient for each scale compared to the median SPV of all five tools (reference standard). The color code represents the ratio of the number of PIMs identified by a given scale to the total number of medications taken, where “0” being the least PIM identified by the tool to “1” being the highest PIM identified. PIM, potentially inappropriate medication; SPV, standardized PIM value.

A total of 2993 medications were prescribed to 467 patients at the time of geriatric assessment. The prevalence of PIM use according to Beers Criteria, STOPP/START criteria, PRISCUS list, FORTA and the EU‐7 PIM list was found to be 364 (77.94%), 210 (55.03%), 288 (61.80%), 393 (84.51%), and 333 (71.30%) respectively. The Bland–Altman plots in Figure 2 show the performance of the PIM assessment tools with respect to the “gold standard” (Figure 2A–E). Bias is a measure of the disagreement between a given scale and the reference standard, lower bias indicating better agreement. Thus, the EU (7)‐PIM list, with the least bias of 0.010 had the highest agreement with the reference standard. The width of the 95% CI was also the narrowest for EU (7)‐PIM list, suggesting good agreement between the SPVs obtained from the scale and the median (Table S1). These findings were further corroborated by Lin's Concordance coefficient of 0.76 (Precision *ρ* = 0.76 and Accuracy *χ*_a_ of 0.99) (Table S2). The PRISCUS list showed the next best agreement based on the observed bias followed by the Beer's criteria (Bias = 0.035 and −0.039, respectively). FORTA showed the highest bias and least agreement with the median SPV. Notably, Beer's and FORTA overestimated the PIM whereas STOPP and START, PRISCUS and EU^7^ PIM list underestimated the PIMs. Figure 3 shows the heatmap of SPV obtained from each scale and the reference standard for each patient, color coded according to the value of SPV. Visual check of the heatmap again shows close agreement between EU^7^ PIM list and the reference standard and highlights the significant overestimation of PIM by FORTA and to a lesser extent by Beer's (it may be noted that the color code in the heatmap corresponds to higher values of SPV as compared to the reference standard). Likewise, it is also obvious from the heatmap that STOPP and START significantly underestimates PIM followed by PRISCUS to a lesser extent.

### 3.3 Covariates and PIM use

Univariate analysis showed that hypertension, diabetes, and polypharmacy to be significantly associated with PIM use (*p* < 0.05). However, in the multivariate analysis using logistic regression, only polypharmacy was found to be significantly associated with PIM use (OR = 1.47 (1.33–1.63); *p* < 0.001) (Table 2).

**TABLE 2:** Results of univariate and multivariate regression analysis (*n* = 467).

				Univariate			Multivariate		
		Patients with no PIM use (*n* = 111)	Patients with PIM use (*n* = 356)	OR	95% CI	*p*‐value	OR	95% CI	*p*‐value
Age group	≤70	53 (47.7)	169 (47.5)	1.01	0.66–1.55	0.959			
	>70	58 (52.3)	187 (52.5)						
Sex	Male	92 (82.9)	280 (78.7)	1.31	0.75–2.29	0.334			
	Female	19 (17.1)	76 (21.3)						
Comorbidities	HTN	54 (48.6)	174 (48.9)	1.01	0.66–1.55	0.967			
	DM	20 (18.0)	112 (31.5)	2.09	1.23–3.56	0.006	0.833	0.46–1.50	0.54
	COPD	8 (7.2)	56 (15.8)	2.41	1.11–5.23	0.022	0.851	0.36–1.97	0.70
	Cardiovascular disease	14 (12.6)	67 (18.8)	1.61	0.86–2.99	0.132			
No. of medications	>5	32 (28.8)	244 (68.5)	5.38	3.37–8.59	0.000	1.463	1.32–1.61	<0.001
Impaired renal function	CrCl < 30	6 (5.5)	11 (3.1)	1.93	0.69–5.4	0.625			

*Note*: Nagelkerke R square: 28%.

Abbreviations: CI, Confidence interval; COPD, Chronic Obstructive Pulmonary Disease; CrCl, Creatinine Clearance; DM, Diabetes Mellitus; OR, Odds ratio.

## 4 DISCUSSION

PIMs in older patients with cancer has become a matter of concern over the years. Several tools are available for the identification of PIM, each with a different approach and relevance. However, very few studies have reported the utility of these tools in the geriatric oncology population.^22^, ^23^ One of the main limitations of these tools is that most of them were developed following country‐specific guidelines, national drug markets, and prescribing habits. Therefore, their transferability across countries is uncertain.^24^ Thus, the purpose of this study was to identify the most suitable tool in identifying PIM in the geriatric oncology clinic at a tertiary care cancer hospital in India. In general, wide variation was observed in the accuracy of these tools which underscored the poor concordance between EU(7)‐PIM list, Beer's criteria, STOPP criteria, Chinese criteria, PRISCUS list, and FORTA reported by earlier investigators.^25^, ^26^, ^27^ We also identified polypharmacy as the single most important determinant of PIM use.

We observed that the EU (7)‐PIM list had higher agreement with the reference standard, followed by PRISCUS list and Beer's criteria, in identifying PIMs in older Indian patients with cancer. It is pertinent to note that in the development of EU(7)‐PIM list, several international PIM lists including the German PRISCUS list, the Beer's criteria, the Canadian list, and the French list along with the drugs suggested by experts on prescribing elderly patients from multiple European nations were considered.^28^ Understandably, it performed better than other tools. Further, a major advantage with EU^7^ is that it can be used even when minimal clinical information is available. We observed a relatively higher bias when FORTA and STOPP/START criteria was used for PIM identification. These tools were developed for practical utility and usability and therefore include only a limited number of drugs.^29^, ^30^ Several covariates (gender, polypharmacy, comorbidities, etc.) are known to affect PIM use.^31^, ^32^, ^33^, ^34^ Multiple reports show that patients having more than five concomitant medications have been found to have increased PIM use, which is in line with our results.^35^, ^36^, ^37^, ^38^, ^39^, ^40^

There is no “gold standard” tool to assess PIMs. Each scale has its own unique characteristics. In order to overcome varying sensitivity and specificity of these tools, several studies have utilized two or three PIM assessment tools together.^35^, ^41^, ^42^, ^43^ However, it is not a sustainable solution, particularly in busy clinics, in the long run. Therefore, identification of the single most reliable tool that can be applied in geriatric oncology setting is extremely important. Since polypharmacy is very common in oncology, an accurate PIM identification tool coupled with thorough medication reconciliation aided by deprescribing guidelines can improve clinical outcomes in older cancer patients.

Our study had a few limitations. As discussed earlier, EU^7^ is a comprehensive scale for PIM assessment. It might be the most reliable among available tools in the context of geriatric oncology practice in India as our findings has indicated. However, its sensitivity and specificity for identifying PIM in this setting is still unknown. Our study was not designed to answer that question. Future studies should address this gap and determine whether the EU^7^ is optimal or if there is need to develop a country specific scale. It is pertinent to note that EU,^7^ FORTA and PRISCUS are region specific tools designed to suit local prescription practices. Also, prospective studies should be carried out in older cancer patients to evaluate the impact of EU^7^ based evaluation of PIM on various health outcomes such as hospitalization, mortality, and functional decline.

## 5 CONCLUSION

A high degree of discordance was observed between the tools. The EU (7)‐PIM list was found to have the least bias and therefore may be considered the most reliable among all tools studied. Future studies should determine its sensitivity and specificity, and further optimize the tool as per local requirements, to aid better clinical decision‐making in Indian geriatric oncology practice.

## AUTHOR CONTRIBUTIONS

**Sharath Kumar:** Data curation (equal); formal analysis (equal); writing – original draft (equal). **Renita Castelino:** Data curation (equal); formal analysis (equal); writing – original draft (equal). **Abhijith Rao:** Data curation (equal). **Shreya Gattani:** Data curation (equal). **Anita Kumar:** Data curation (equal). **Anupa Pillai:** Data curation (equal). **Arshiya Sehgal:** Data curation (equal). **Pallavi Rane:** Formal analysis (equal). **Anant Ramaswamy:** Data curation (equal); visualization (equal). **Ratan Dhekale:** Data curation (equal). **Jyoti Krishnamurthy:** Data curation (equal). **Shripad Banavali:** Resources (equal). **Rajendra Badwe:** Resources (equal). **Kumar Prabhash:** Conceptualization (equal); supervision (equal); visualization (equal). **Vanita Noronha:** Conceptualization (equal); supervision (equal); visualization (equal); writing – review and editing (equal). **Vikram Gota:** Conceptualization (equal); supervision (equal); visualization (equal); writing – review and editing (equal).

## CONFLICT OF INTEREST STATEMENT

There are no conflicts of interest.

## Supporting information


Table S1.
Table S2.

## Data Availability

All data generated or analyzed during this study are included in this article. The individual de‐identified participant data collected, study protocol, and the statistical analysis plan are available after publication without an end date, from the corresponding author on reasonable request.
