# Supplementary material for: Performance of potentially inappropriate medications assessment tools in older Indian patients with cancer
Source: Cancer Med. 2024 Jan 6;13(1):e6797. doi: 10.1002/cam4.6797 (PMC10807583; doi:10.1002/cam4.6797)
Supplement: Supplementary file 1 — Table S1. Table S2. [file CAM4-13-e6797-s001.docx]

**Supplementary table 1: Bias estimates and the limits of agreement of different PIM tools compared with the reference standard given by the Bland-Altman Plots.**

|  | **Reference standard versus** | | | | | | | | | |
| --- | --- | --- | --- | --- | --- | --- | --- | --- | --- | --- |
|  | **AGS Beer’s Criteria** | | **STOPP and START Criteria** | | **PRISCUS List** | | **FORTA List** | | **EU (7)-PIM List** | |
| **(n=467)** | Est. | 95% CI | Est. | 95% CI | Est. | 95% CI | Est. | 95% CI | Est. | 95% CI |
| **Bias** | -0.039 | (-0.053 to -0.025) | 0.076 | (0.060 to 0.092) | 0.035 | (0.021 to 0.049) | -0.148 | (-0.165 to -0.130) | 0.010 | (-0.001 to 0.022) |
| **Lower LoA** | -0.336 | (-0.359 to -0.312) | -0.272 | (-0.300 to -0.244) | -0.261 | (-0.285 to -0.237) | -0.522 | (-0.552 to -0.493) | -0.250 | (-0.270 to -0.229) |
| **Upper LoA** | 0.257 | (0.234 to 0.281) | 0.425 | (0.398 to 0.453) | 0.332 | (0.308 to 0.356) | 0.226 | (0.196 to 0.255) | 0.271 | (0.251 to 0.292) |
| **Width of LoA** | 0.593 | - | 0.697 | - | 0.53 | - | 0.748 | - | 0.521 | - |

**Est. = Estimate, LoA = Limit of Agreement**

**Supplementary Table 2: Agreement between Median SPV and different PIM tools by Lin's Concordance Index**

| **Evaluation parameters** | **Beer’s Criteria** | **STOPP/START** | **PRISCUS** | **FORTA** | **EU (7)** |
| --- | --- | --- | --- | --- | --- |
| Precision ρ | 0.7081 | 0.520 | 0.683 | 0.680 | 0.762 |
| Scale Shift ω | 1.104 | 0.932 | 1.053 | 1.359 | 1.042 |
| Effect Size υ | 0.193 | 0.418 | 0.190 | 0.665 | 0.056 |
| Accuracy χ_a_ | 0.976 | 0.917 | 0.980 | 0.787 | 0.997 |
| Lin's Coefficient of Concordance ρ_c_ | 0.691 | 0.477 | 0.670 | 0.536 | 0.760 |
| 95% CI | 0.641 – 0.734 | 0.415 – 0.545 | 0.612 – 0.710 | 0.488 – 0.584 | 0.721 – 0.796 |
